# Genomic evolution of BA.5.2 and BF.7.14 derived lineages causing SARS-CoV-2 outbreak at the end of 2022 in China

**DOI:** 10.3389/fpubh.2023.1273745

**Published:** 2023-11-30

**Authors:** Wentao Zhu, Xiaoxia Wang, Yujin Lin, Lvfen He, Rui Zhang, Chuan Wang, Xiong Zhu, Tian Tang, Li Gu

**Affiliations:** ^1^Department of Infectious Diseases and Clinical Microbiology, Beijing Institute of Respiratory Medicine and Beijing Chao-Yang Hospital, Capital Medical University, Beijing, China; ^2^Central and Clinical Laboratory of Sanya People’s Hospital, Sanya, Hainan, China; ^3^West China School of Public Health and West China Fourth Hospital, Sichuan University, Chengdu, Sichuan, China; ^4^Department of Laboratory Medicine, Beijing Chao-Yang Hospital, Capital Medical University, Beijing, China

**Keywords:** SARS-CoV-2, lineage, selection pressure, mutation, China

## Abstract

Since the end of 2022, when China adjusted its COVID-19 response measures, the SARS-CoV-2 epidemic has rapidly grown in the country. It is very necessary to monitor the evolutionary dynamic of epidemic variants. However, detailed reports presenting viral genome characteristics in China during this period are limited. In this study, we examined the epidemiological, genomic, and evolutionary characteristics of the SARS-CoV-2 genomes from China. We analyzed nearly 20,000 genomes belonging to 17 lineages, predominantly including BF.7.14 (22.3%), DY.2 (17.3%), DY.4 (15.5%), and BA.5.2.48 (11.9%). The Rt value increased rapidly after mid-November 2022, reaching its peak at the end of the month. We identified forty-three core mutations in the S gene and forty-seven core mutations in the ORF1ab gene. The positive selection of all circulating lineages was primarily due to non-synonymous substitutions in the S1 region. These findings provide insights into the genomic characteristics of SARS-CoV-2 genomes in China following the relaxation of the ‘dynamic zero-COVID’ policy and emphasize the importance of ongoing genomic monitoring.

## 1 Introduction

The coronavirus disease 2019 (COVID-19) pandemic, caused by the severe acute respiratory syndrome coronavirus 2 (SARS-CoV-2), has been ongoing for over three years, posing an unprecedented challenge to global public health (1). As of August 1, 2023, there have been over 768 million confirmed cases and approximately 7 million cumulative deaths recorded as a result of SARS-CoV-2 infection (https://covid19.who.int/). Moreover, the number of patients infected with the virus continues to rise (2). The genome of SARS-CoV-2 undergoes mutations during viral replication, leading to the emergence of mutated viruses that are subjected to selective pressures. During its rapid spread and explosive radiation, SARS-CoV-2 has accumulated mutations on a genome-wide scale, continuously evolving into new variants that spread quickly to different parts of the world (3–6). To date, five variants of concern (VOCs) have emerged and been designated for monitoring: alpha, beta, gamma, delta, and omicron (7). Since its emergence in November 2021, the Omicron variants has acquired a significant number of new mutations, with 80% of them accumulating in the S protein. This has resulted in the development of more than 700 Omicron lineages, including five main lineages (BA.1, BA.2, BA.3, BA.4, and BA.5) (8). The Omicron variant, with over 30 mutations in the S protein, has spread worldwide within a few months. It is believed to be more infectious and capable of immune escape than previous VOCs (9, 10). The evolution of SARS-CoV-2 is continuing, which leads to the expected generation of new variants. As of July 30, 2023, two Variants of Interest (VoIs) (i.e., XBB.1.5 and XBB.1.16) and seven Variants under Monitoring (VuMs) (i.e., BA.2.75, CH.1.1, XBB, XBB.1.9.1, XBB.2.3, and EG.5) were listed by the World Health Organization (WHO) (https://www.who.int/publications/m/item/weekly-epidemiological-update-on-covid-19---3-august-2023).

Variants of SARS-CoV-2, especially those linked to epidemiological events, are currently being closely monitored by the WHO and other public health agencies worldwide (11). Whole-genome sequencing (WGS) greatly aids in tracking viral genomic changes and helps in comprehending phenotypic changes (12). In the last three years, a significant volume of genomic data has been generated, informing local and international communities about crucial aspects of the pandemic. This data has served as a foundation for adjusting prevention and control strategies (13).

At the end of 2022, China discontinued the ‘dynamic zero-COVID’ policy (14). Subsequently, China has entered a new phase, where the number of people infected with Omicron has surged (15). To monitor the evolutionary process of epidemic variants in China is very important (16). This study primarily concentrates on examining the genomic and evolutionary features of SARS-CoV-2 genomes in China during this period.

## 2 Materials and methods

### 2.1 Sample selection and nucleic acid test

From December 2022 to January 2023, nasopharyngeal samples were collected from individuals at the Clinical Laboratory of Sonya People’s Hospital and preserved in 3 mL of inactivated viral sample preservation solution. A 200 μL sample was taken from each specimen to extract total RNA using the QIAamp Viral RNA Mini Kit (Qiagen, Germany), which was then eluted with RNase-free water. The concentration of total RNA was determined using the Qubit 2.0 fluorometer (Invitrogen, United States). The clinical one-step real-time PCR was conducted to detect SARS-CoV-2 by targeting the ORF1ab and N genes, respectively. This was done using the 2019-nCoV detection kit (PCR-Fluorescence) in accordance with the manufacturer’s instructions (17). The total RNA from positive samples was stored at −80°C until further analysis.

### 2.2 Whole-genome sequencing and assembly

The total RNA from SARS-CoV-2 positive samples with a Ct value of 35 or less in both the ORF1ab and N genes was randomly selected for next-generation sequencing (NGS). The library was prepared using the ATOPlex RNA Multiplex PCR-based Library Preparation Set V3.1, following the manufacturer’s instructions (940–000132-00, MGI, China). The process included reverse transcription, purification, end repair, and adaptor addition. The obtained library underwent further processing using the DNBSEQ One-step DNB Preparation Kit (1,000,026,466, MGI, China) and was subsequently sequenced on the MGISEQ-2000RS platform using SE100 technology.

The raw data (fastq reads) from each sample were filtered using FastQC tool v0.11.9 (18). Trimmomatic v0.39 (19) was then used to remove adapter sequences and low-quality base calls (Q < 30). The filtered reads, which were mapped to the Wuhan-Hu-1 genome (NC_045512.2), were utilized to acquire the consensus sequence for each sample through SPAdes v3.15.4 (20).

### 2.3 Variant calling and phylogenetic analysis

All complete SARS-CoV-2 genomes that belonged to the dominant lineages circulating in mainland China after the implementation of 10 new measures were downloaded from the GISAID and NCBI database (as of April 27, 2023). Genomes with incomplete collection dates and low coverage were excluded. The PANGO lineages of all genomes were determined according to the pangolin nomenclature. An in-house script was used to parse genomes with “N” and low coverage in order to identify the single nucleotide polymorphisms (SNPs) (21). The mutations in each genome were identified using Wuhan-Hu-1 as the reference. The frequency of each variant site was calculated by dividing the number of genomes containing the site by the total number of genomes in the lineage. A mutation with a frequency between 0.8 and 1.0 was considered a core mutation. The alignment was obtained using Nextalign, and phylogenetic analysis was performed using Nextstrain pipelines v12 under the SARS-CoV-2 workflow (22). The resulting tree was visualized using the online tool auspice (https://auspice.us/).

### 2.4 Non-synonymous (Ka) and synonymous (Ks) calculation

The nucleotides and amino acids from ORFs of each genome were predicted and obtained using ORFfinder (https://www.ncbi.nlm.nih.gov/orffinder/). The protein-coding DNA alignments were constructed using Parallel Alignment and Back-Translation (ParaAT v2.0) (23). The rates of non-synonymous substitutions (Ka), synonymous substitutions (Ks), and Ka/Ks ratio between the subject genome and the reference genome were calculated using the PAML-yn00 pipeline with the Yang and Nielsen (YN) method (24).

### 2.5 Statistical analysis

The instantaneous effective reproduction number (*Rt*) is estimated using the R package EpiEstim v0.1 (25). The estimation is based on the number of genomes reported per day, with generation time of 3.0 days and incubation periods of 4.0 days (26). Statistical plots were generated using Origin Pro 2021 version. The significance of differences was assessed using the χ^2^, Mann–Whitney U test, or Wilcoxon test, with *p* < 0.05 indicating statistical significance.

## 3 Results

### 3.1 Genomic epidemiology of variants circulating in China

**Figure 1:**
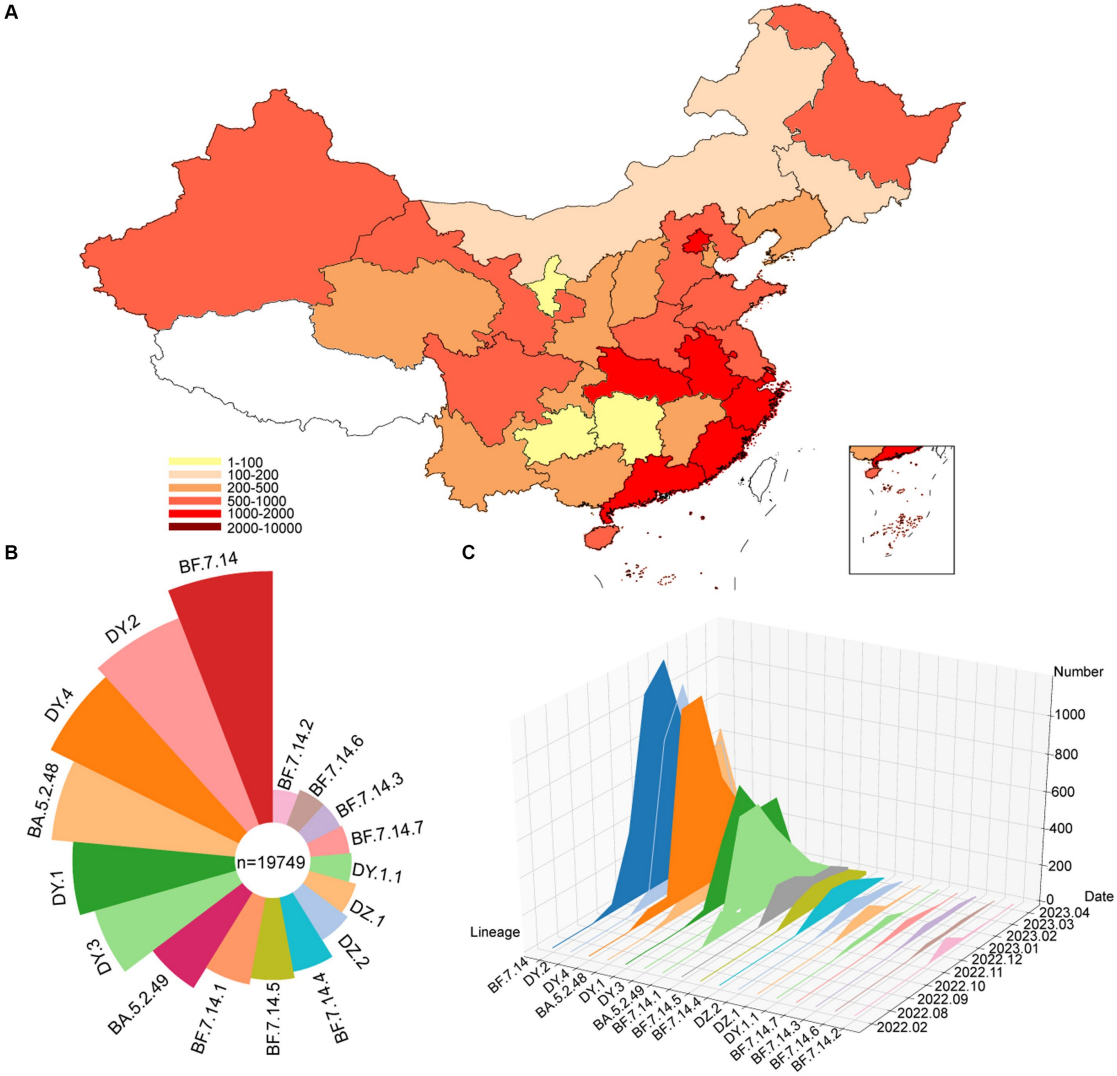
Temporal contribution of circulating SARS-CoV-2 lineages after ending the dynamic zero-COVID policy. **(A)** Map showing the genome contribution in Chinese mainland. The number of SARS-CoV-2 genomes are defined by the color-bar. **(B)** Overall SARS-CoV-2 lineages contribution in Chinese mainland. The lineages are represented by corresponding colors. The size represents the proportion of cases of each lineage out of 19,749 genomes. **(C)** The genome number of each circulating SARS-CoV-2 lineages changes over month. A total of 17 circulating lineages are presented from first detected to April 2023.

From December 2022 to January 2023, specimens that tested positive for SARS-CoV-2 were collected in Sonya, of which 90 specimens were randomly selected for whole genome sequencing. To analyze the genomic epidemiology after ending the zero-COVID policy in the Chinese mainland, the genomes of circulating lineages and their corresponding metadata were downloaded from the GISAID and NCBI database. A total of 19,749 genomes, including those from this study, were used to investigate genomic epidemiology. The study showed that these viruses were prevalent in 30 provincial-level administrative regions in the Chinese mainland (Figure 1A), with the highest number of genomes reported in Beijing (1640), Chongqing (1575), Fujian (1506), Gansu (1280), Guangdong (1122), and Guangxi (1017). The dominant pangolin lineages were composed of BF.7.14 (4,396, 22.3%), DY.2 (3,425, 17.3%), DY.4 (3,070, 15.5%), BA.5.2.48 (2,358, 11.9%), DY.1 (1832, 9.3%), and DY.3 (1,505, 7.6%) (Figure 1B and Supplementary Figure S1). The number of confirmed cases in the SARS-CoV-2 outbreak increased rapidly from December 2022 to February 2023, with most new additions in January 2023 (Figure 1C and Supplementary Figure S1). The number of new cases decreased to low levels in April 2023. The frequencies of the different lineages varied across regions.

**Figure 2:**
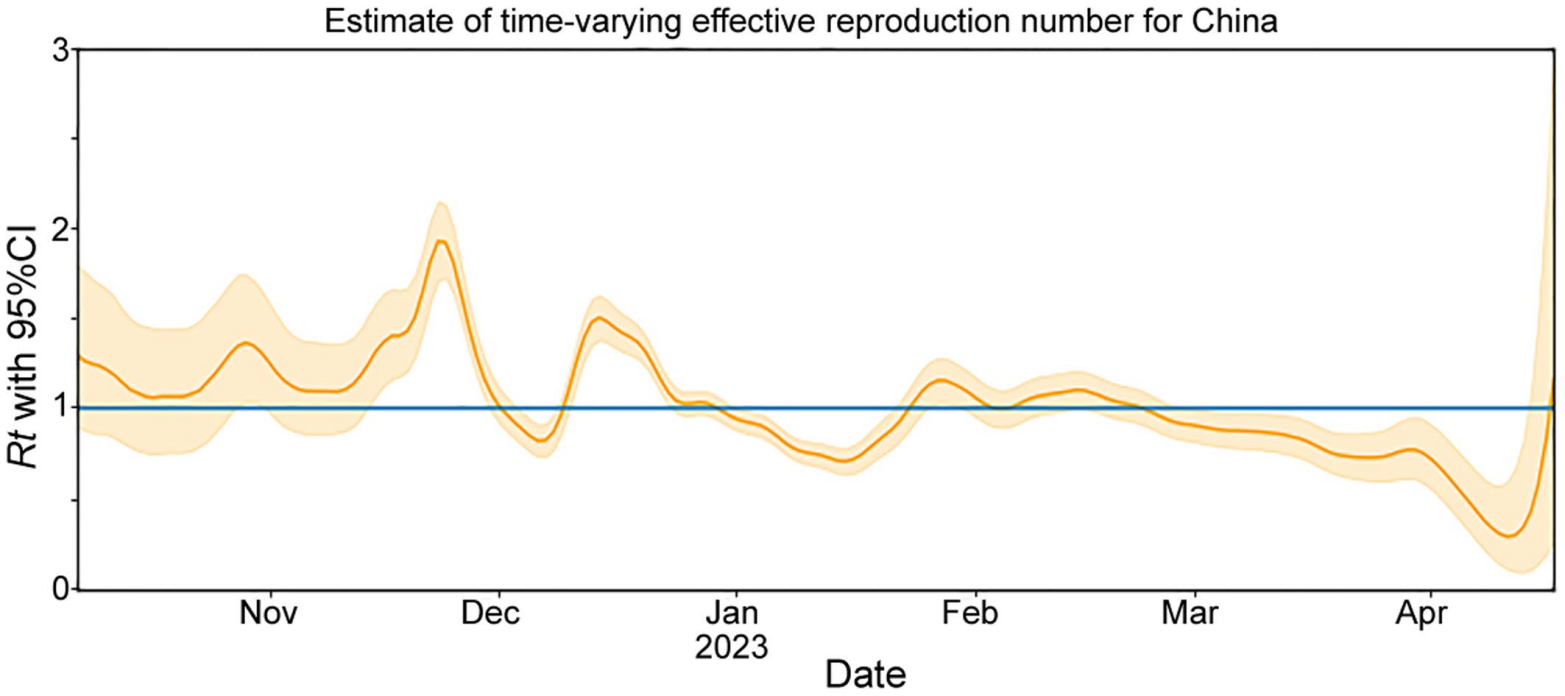
The effective reproduction number (*Rt*) for COVID-19 in China during November 2022 to April 2023. The solid yellow line represents the maximum likelihood estimates. The yellow background indicates their 95% confidence intervals (CI) based on the 2.5% quantile and 97.5% quantile. The blue line represents *Rt* = 1.

The *Rt* is estimated based on the number of genomes reported per day (Figure 2). From October to mid-November 2022, the circulating lineages (BA.5.2, BF.7.14 and their descendant lineages) were observed in China and underwent local transmission. The estimated *Rt* was increased rapidly after mid-November 2022, and peaked at the end of the month. However, there was an unexpected decrease in the *Rt*, which fell below 1 in early December, which may result from under-sequencing of SARS-CoV-2 during this period. Nevertheless, a second peak was observed in mid-December. From January to April, 2023, *Rt* decreased gradually under 1 and showed a fluctuation around 1 during late January and early February.

### 3.2 Evolutionary relationship

**Figure 3:**
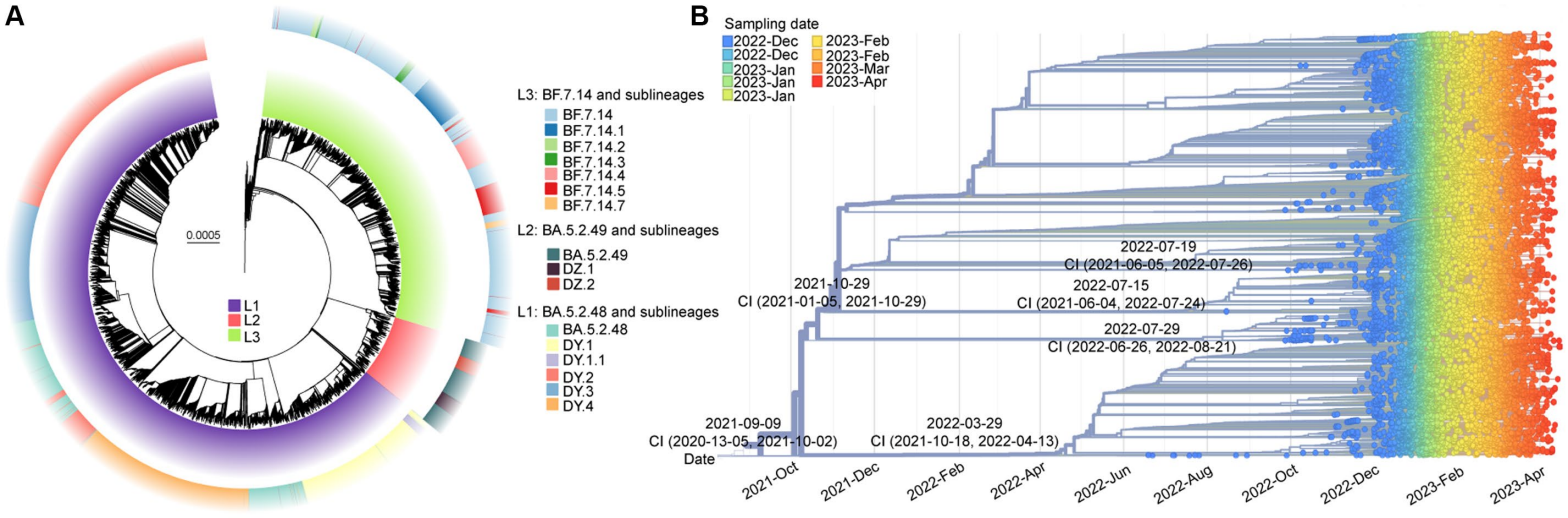
The molecular evolutionary relationships of circulating SARS-CoV-2 lineages during ending the dynamic zero-COVID policy. **(A)** The Maximum likelihood phylogenetic tree based on 19,749 SARS-CoV-2 genomes with Wuhan-Hu-1 as reference. The lineages and its descendant lineages were colored with corresponding colors. **(B)** The Nextstrain’s phylodynamic analysis. The circle with different colors were labeled with sampling date. The phylogenetic tree was visualized by the Auspice online tool. The tMRCA with confidence interval (CI) are labeled on the branches.

After removing low-quality genomes, a total of 10,474 SARS-CoV-2 genomes were utilized to construct a phylogenetic tree. The results, shown in Figure 3A, indicated that these sequences formed three main clades (L1-L3). Clade L1 represented BA.5.2.48 and its descendant lineages (DY.1, DY.1.1, DY.2, DY.3, and DY.4), L2 represented BA.5.2.49 and its descendant lineages (DZ.1 and DZ.2), and L3 represented BF.7.14 and its descendant lineages (BF.7.14.1, BF.7.14.2, BF.7.14.3, BF.7.14.4, BF.7.14.5, and BF.7.14.7). Except for DY.3, BA.5.2.49, and BF.7.14, all of the lineages, were first detected in the Chinese mainland.^1^

The evolutionary origins of these dominant lineages have been estimated. The temporal signal test results (Supplementary Figure S2) showed a significant correlation between the sampling dates and the root-to-tip distance (*R^2^* = 0.818). The estimated rate was 31.338 substitutions per year (Supplementary Figure S2). According to Figure 3B, the estimated date for the most recent common ancestor (TMRCA) of BF.7.14 and its descendant lineages was March 29, 2022, with a confidence interval of October 18, 2021, to April 13, 2022. The emergence date of BA.5.2.49 and its descendant lineages was inferred to be July 29, 2022, with a confidence interval from June 26, 2022, to August 21, 2022. The projected date for the emergence of DY.4 is estimated to be July 19, 2022, with a confidence interval from June 5, 2021, to July 26, 2022.

### 3.3 Mutational analysis

**Figure 4:**
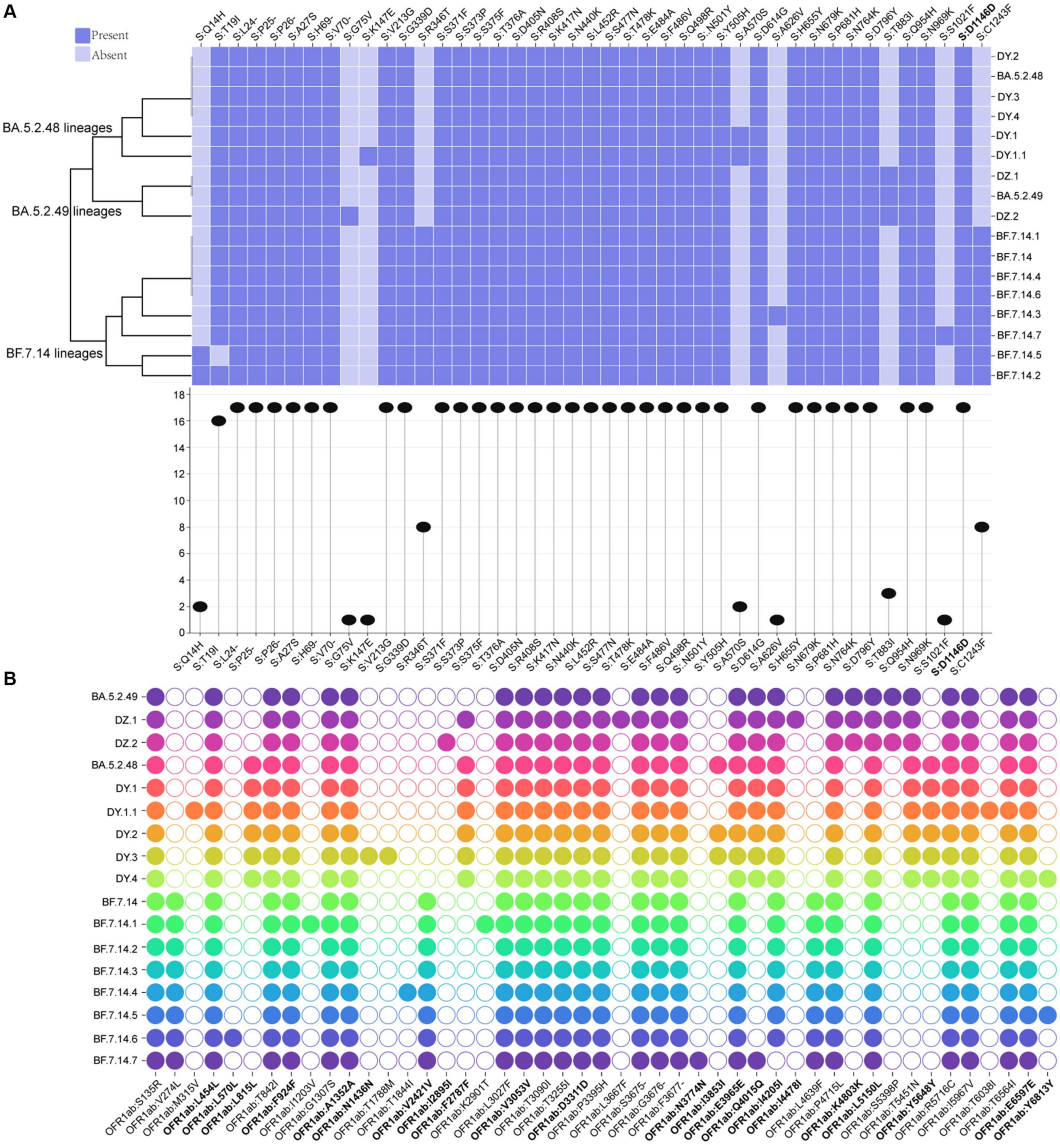
Core amino acid substitutions. **(A)** The core amino acid substitutions are presented or absented in S proteins of all circulating lineages. The substitution profiles are clustered by lineages. The plot on the bottom indicates the frequencies of individual substitutions across all lineages. **(B)** The core amino acid substitutions are presented or absented in ORF1ab of all circulating lineages. The substitutions with bold represented synonymous substitutions.

A total of 43 core mutation profiles were detected in S proteins, including five deletions and one synonymous mutation (Figure 4A). Each lineage shared 33 core mutations, with 34–37 mutations in the S protein, respectively, indicating that the mutation frequency of the other 10 core mutations varied across lineages (Figure 4A). The BF.7.14 and its descendant lineages had R346T and C1243F mutations in the S protein, which was absent in the BA.5.2 and its descendant lineages (Figure 4A). The study also identified several unique core mutations in the S protein, including G75V in DZ.2, K147E in DY.1.1, A626V in BF.7.14.3, and S1021F in BF.7.14.7. The T883I mutation was unique to the S protein of BA.5.2.49 and its descendant lineages, compared to BA.5.2.48 and its descendant lineages, as well as BF.7.14 and its descendant lineages.

Meanwhile, 47 core mutation profiles were identified in the ORF1ab gene, including twenty synonymous mutations and one deletion (Figure 4B). All circulating lineages shared 22 core mutations, with 26–31 core mutations in the ORF1ab (Figure 4B). BF.7.14 and its descendant lineages contained three marker mutations in the ORF1ab gene: V274L, V2421V, and L4639F, which were distinct from those found in BA.5.2 and its descendant lineages. Unique mutations (L815L, F2787F, and Y5648Y) in the ORF1ab gene were presented in all BA.5.2.48 and its descendant lineages, but were absent in the other two lineages (BF.7.14 and BA.5.2.49) and their corresponding descendant lineages (Figure 4B). The synonymous mutation (K4803K) in ORF1ab of BA.5.2.49 and its descendant lineages was a distinct mutation when compared to that of BA.5.2.48 and its descendant lineages, as well as BF.7.14 and its descendant lineages. Additionally, unique mutations were found only in certain lineages, including M315V and T6038I for DY.1.1, L570L for BF.7.14.6, I1203V and K2901T for BF.7.14.1, N1436N and T1788M for DY.3, I2895I for DZ.2, L3667F and I4478I for DZ.1, and N3774N for BF.7.14.7.

### 3.4 Selection analysis

**Figure 5:**
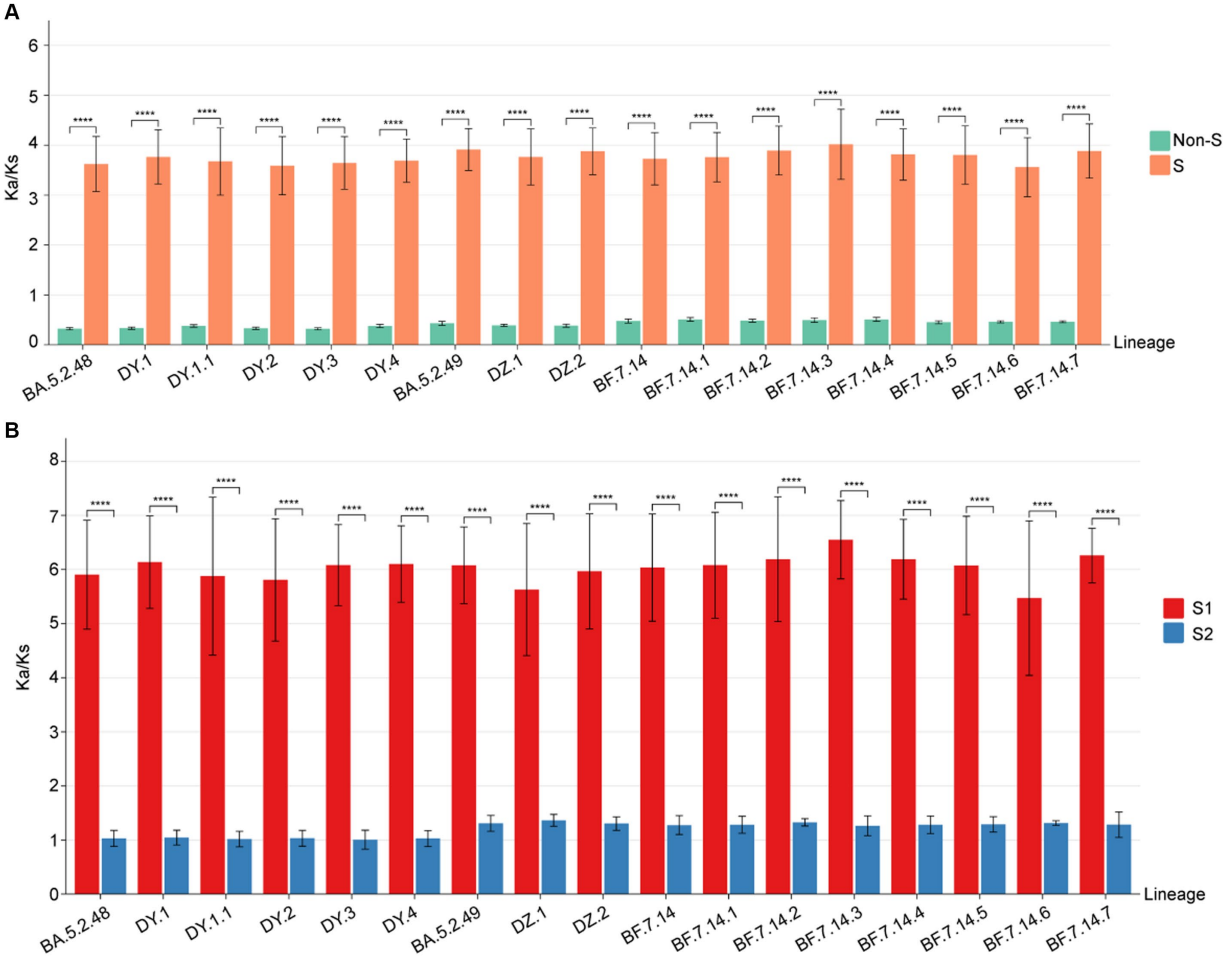
The Ka/Ks value comparison among all circulating lineages. **(A)** The Ka/Ks values of S and non-S genes. The Ka/Ks values are significantly higher for the S gene than non-S genes of all circulating lineages (*p* < 0.05). **(B)** The Ka/Ks values of S1 and S2 regions. The Ka/Ks values are significantly higher for the S1 region than S2 region of all circulating lineages (*p* < 0.05).

To identify positive selection signals in the ongoing evolution of SARS-CoV-2, we utilized the high-quality SARS-CoV-2 genomes to calculate the Ka (non-synonymous substitutions per non-synonymous site) and Ks (synonymous substitutions per synonymous site) values between the genomes from different lineages and the reference genome (Wuhan-Hu-1, NC_045512.2). The results (Figure 5A) revealed that the Ka/Ks value for the S gene in all lineages was significantly higher than 1, with a median ranging from 3.80 to 4.10 (Supplementary Table S1). On the other hand, the Ka/Ks value for the remaining concatenated (non-S) genes was significantly less than 1 for all lineages, with the median ranging from 0.31 to 0.52 (Supplementary Table S1). Furthermore, the Ka/Ks value for the S gene was significantly higher (*p* < 0.001) than that of the non-S gene in all circulating lineages. This suggests that positive selection is the primary evolutionary force driving the evolution of the S protein, while purifying selection is acting on the protein sequences of the non-S genes in all circulating SARS-CoV-2 (Figure 5A).

To determine if selective pressure is consistent across all regions of the S gene, we calculated the Ka/Ks values for both the S1 and S2 regions in comparison to the reference genome. The Ka/Ks values for the S1 region of all circulating lineages were significantly higher than 1, with a median range from 6.22 to 6.67 (Supplementary Table S1). The Ka/Ks values for the S2 region of all circulating lineages were slightly higher than 1, with a median range from 1.05 to 1.37 (Supplementary Table S1). Importantly, the Ka/Ks values for the S1 region of all circulating lineages were significantly higher than those of the S2 region (*p* < 0.01) (Figure 5B). These results indicated that the positive selections of all circulating lineages were primarily derived from non-synonymous substitutions in the S1 region. Additionally, the corresponding S2 regions were under slightly positive selections overall (Figure 5B).

## 4 Discussion

Our findings indicate that the prevailing variants were primarily derived from BA.5.2 and BF.7 lineages. The diversity of lineages varied among provinces and cities, suggesting regional differences in pandemic patterns. This variation in transmission advantage between BA.5.2 and BF.7 lineages is supported by different regions exhibiting distinct symptom profiles (27–29). Prior to December 2022, sporadic occurrence of BA.5.2 and its descendant lineages as well as BF.7 and its descendant lineages, was observed in China (30). This suggests that these occurrences reflected broader epidemiological patterns prevailing in China before this period. The number of new cases continued to rise rapidly throughout December 2022, peaking in January 2023, before declining from February 2023 onwards. BA.5.2- and BF.7-derived lineages showed fitness levels approximately 24 and 20 times higher than the prototype, which may explain their surge in this period (31).

The abundance of SARS-CoV-2 genomes presents a unique opportunity to infer the virus’s evolutionary dynamics. The non-synonymous mutations made up the top viral mutations with most accumulated in the S and ORF1ab proteins, indicating local evolution and subsequent adaptation. The emergence date of these novel lineages was estimated based on the significant correlation between sampling dates and the root-to-tip distance, which provides information about the origin of SARS-CoV-2 lineages. Additionally, low-frequency mutations have been found throughout the entire genome of all circulating lineages, confirming the constant mutation of the virus and suggesting the potential emergence of new lineages.

The S protein binds to the host cell receptor angiotensin-converting enzyme 2 (ACE2), which determines viral cell entry. This process can be facilitated by the activation of the TM protease serine 2 (TMPRSS2) (32). Analysis of selection pressure has revealed a significant positive selection in the S protein, specifically in the S1 region. This implies that SARS-CoV-2 has undergone rapid evolution as a result of the ongoing evolutionary arms race between viruses and hosts (33). It is important to note that due to the variations in fitness and other factors, such as imported cases, new lineages of SARS-CoV-2 may emerge from existing ones through positive selection, rendering older lineages obsolete and potentially leading to new risks for human health.

In conclusion, the outbreak witnessed a rapid and substantial rise in SARS-CoV-2 infected cases as a result of the co-circulation of BF.7-derived and BA.5.2-derived lineages. It is imperative to consistently carry out extensive genomic monitoring to effectively track and understand the dynamics of SARS-CoV-2.

## Data availability statement

The datasets presented in this study can be found in online repositories. The names of the repository/repositories and accession number(s) can be found at: [https://www.gisaid.org/, EPI_ISL_17805437 to EPI_ISL_17805525].

## Ethics statement

The studies involving humans were approved by Ethics Committee of Sonya People’s Hospital (NO: SYPH-2022-043). The studies were conducted in accordance with the local legislation and institutional requirements. Written informed consent for participation was not required from the participants or the participants' legal guardians/next of kin in accordance with the national legislation and institutional requirements.

## Author contributions

WZ: Conceptualization, Data curation, Formal analysis, Methodology, Visualization, Writing – original draft. XW: Data curation, Software, Visualization, Writing – original draft. YL: Resources, Writing – original draft. LH: Resources, Writing – original draft. RZ: Writing – review & editing. CW: Methodology, Writing – review & editing. XZ: Writing – review & editing. TT: Writing – review & editing. LG: Conceptualization, Writing – review & editing.

## Footnotes

^1^ https://github.com/cov-lineages/pango-designation/blob/master/lineages.csv
