# Corrigendum: Genomic evolution of BA.5.2 and BF.7.14 derived lineages causing SARS-CoV-2 outbreak at the end of 2022 in China

**DOI:** 10.3389/fpubh.2024.1397325

**Published:** 2024-03-22

**Authors:** Wentao Zhu, Xiaoxia Wang, Yujin Lin, Lvfen He, Rui Zhang, Chuan Wang, Xiong Zhu, Tian Tang, Li Gu

**Affiliations:** ^1^Department of Infectious Diseases and Clinical Microbiology, Beijing Institute of Respiratory Medicine and Beijing Chao-Yang Hospital, Capital Medical University, Beijing, China; ^2^Central and Clinical Laboratory of Sanya People's Hospital, Sanya, Hainan, China; ^3^West China School of Public Health and West China Fourth Hospital, Sichuan University, Chengdu, Sichuan, China; ^4^Department of Laboratory Medicine, Beijing Chao-Yang Hospital, Capital Medical University, Beijing, China

**Keywords:** SARS-CoV-2, lineage, selection pressure, mutation, China

In the published article, there was an error in affiliations 2 and 3. The correct affiliations appears below.

“^2^Central and Clinical Laboratory of Sanya People's Hospital, Sanya, Hainan, China

^3^West China School of Public Health and West China Fourth Hospital, Sichuan University, Chengdu, Sichuan, China”.

The authors apologize for this error and state that this does not change the scientific conclusions of the article in any way. The original article has been updated.

