# Supplementary material for: Genomic evolution of BA.5.2 and BF.7.14 derived lineages causing SARS-CoV-2 outbreak at the end of 2022 in China
Source: Front Public Health. 2023 Nov 30;11:1273745. doi: 10.3389/fpubh.2023.1273745 (PMC10725193; doi:10.3389/fpubh.2023.1273745)
Supplement: Supplementary file 1 [file Data_Sheet_1.docx]

**Supplementary material:**

Table S1 The Ka/Ks values of dominant circulating lineages

| Lineages | non-S | S | S1 | S2 |
| --- | --- | --- | --- | --- |
| BA.52.48 | 0.31 (0.30-0.33) | 3.80 (3.80-3.80) | 6.22 (6.22-6.22) | 1.05 (1.05-1.05) |
| DY.1 | 0.42 (0.40-0.45) | 3.99 (3.99-3.99) | 6.22 (6.22-6.22) | 1.30 (1.30-1.30) |
| DY.1.1 | 0.48 (0.45–0.50) | 3.91(3.91-3.91) | 6.33 (6.33-6.33) | 1.31 (1.31-1.31) |
| DY.2 | 0.51 (0.49-0.53) | 3.91 (3.91-3.97) | 6.33 (6.33-6.33) | 1.31 (1.31-1.31) |
| DY.3 | 0.48 (0.46-0.50) | 4.03 (4.03-4.03) | 6.56 (6.56-6.56) | 1.31 (1.31-1.31) |
| DY.4 | 0.49 (0.48-0.51) | 4.10 (4.10-4.10) | 6.67 (6.67-6.67) | 1.31 (1.31-1.31) |
| BA.5.2.49 | 0.52 (0.48-0.52) | 3.91 (3.91-3.91) | 6.33 (6.33-6.33) | 1.31 (1.31-1.31) |
| DZ.1 | 0.45 (0.42-0.46) | 3.91 (3.91-3.91) | 6.33 (6.33-6.33) | 1.31 (1.31-1.31) |
| DZ.2 | 0.46 (0.46-0.48) | 3.91 (2.60-3.91) | 6.33 (3.16-6.33) | 1.31 (1.31-1.31) |
| BF.7.14 | 0.46 (0.45-0.48) | 4.10 (4.10-4.10) | 6.33 (3.16-6.33) | 1.37 (1.37-1.37) |
| BF.7.14.1 | 0.37 (0.36-0.40) | 4.04 (2.83-4.04) | 6.67 (6.67-6.67) | 1.05 (1.05-1.05) |
| BF.7.14.2 | 0.33 (0.32-0.34) | 3.85 (3.85-3.85) | 6.32 (6.32-6.32) | 1.05 (1.05-1.05) |
| BF.7.14.3 | 0.33 (0.31-0.34) | 3.80 (3.80 -3.80) | 6.22 (6.22-6.22) | 1.05 (1.05-1.05) |
| BF.7.14.4 | 0.32 (0.30-0.33) | 3.80 (3.80-3.80) | 6.22 (6.22-6.22) | 1.05 (1.05-1.05) |
| BF.7.14.5 | 0.38 (0.35-0.40) | 3.80 (3.80-3.80) | 6.22 (6.22-6.22) | 1.05 (1.05-1.05) |
| BF.7.14.6 | 0.39 (0.37-0.40) | 4.04 (3.85-4.04) | 6.22 (5.95-6.22) | 1.37 (1.37-1.37) |
| BF.7.14.7 | 0.39 (0.36-0.41) | 4.04 (4.04-4.04) | 6.32 (6.32-6.32) | 1.30 (1.30-1.30) |

The Ka/Ks values were calculated as the ratio of the number of nonsynonymous substitutions per nonsynonymous site (Ka) to the number of synonymous substitutions per synonymous site (Ks). The Ka/Ks < 1 indicates negative selection (pure selection). The Ka/Ks > 1 indicates positive Selection (adaptive evolution). Data are median with IQR in following brackets. Non-S, remaining concatenated (except for S) genes; S, spike gene; S1, S1 region; S2, S2 region.


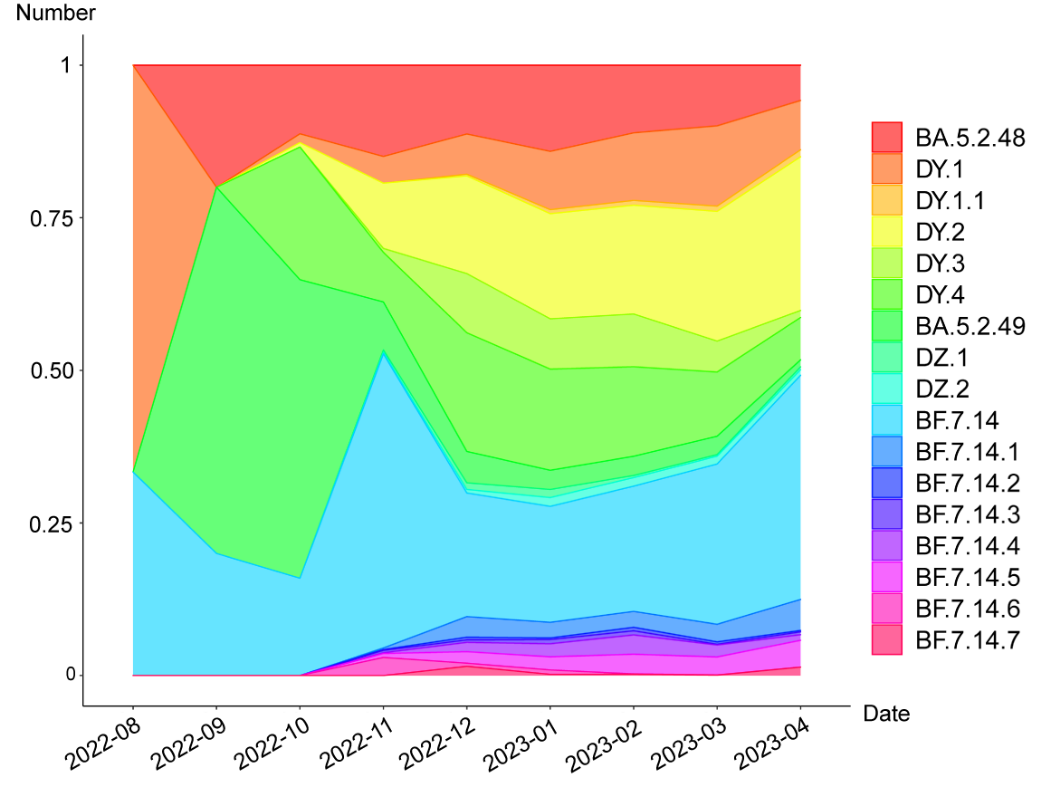


Fig. S1 Trends of all circulating lineages. The lineages are labeled with different colors according to lineages. The Y-axis represents the number of genomes, and the X-axis shows the sampling date.


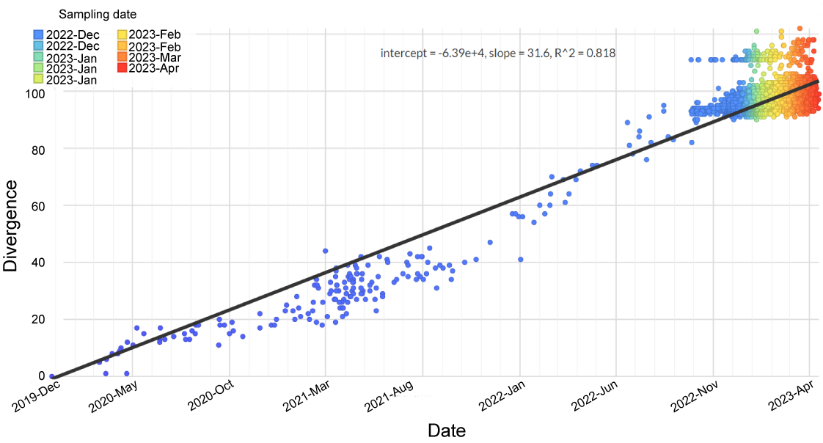


Fig. S2 The correlation of root-tip divergence and sampling date. The Y-axis denoted divergence (the number of mutations in the genome relative to the root), and the X-axis shows the sampling date of each genome. The diagonal line represents average root/tip divergence over time
